# New markers of apoptosis in children on chronic dialysis

**DOI:** 10.1007/s10495-012-0764-8

**Published:** 2012-10-07

**Authors:** Kinga Musiał, Danuta Zwolińska

**Affiliations:** Department of Pediatric Nephrology, Wrocław Medical University, Borowska 213, 50-556 Wrocław, Poland

**Keywords:** Anoikis, E-cadherin, EMMPRIN, MMP-8, Fas, Hsp27

## Abstract

Enhanced apoptosis is characteristic for chronic kidney disease (CKD). A specific type of apoptosis, anoikis, is connected with the extracellular matrix turnover and cell detachment. Although E-cadherin, extracellular matrix metalloproteinase inducer (EMMPRIN) and matrix metalloproteinase (MMP)-8 may play an important role in this process, they have not been analyzed in any nephrological aspect, either in CKD. The aim of study was to evaluate the serum concentrations of E-cadherin, EMMPRIN and their potential regulators (MMP-8, MMP-7, TIMP-1, TIMP-2), with relevance to apoptosis/cell damage markers (sFas, sFasL, Hsp27), in children with CKD. 39 CKD children stages 3–4, 26 CKD children stage 5 still on conservative treatment, 19 patients on hemodialysis (HD), 22 children on automated peritoneal dialysis (APD) and 30 controls were examined. Serum concentrations of those parameters were assessed by ELISA. Median E-cadherin, EMMPRIN and MMP-8 values were significantly increased in patients on dialysis versus those in pre-dialysis period and versus controls. The highest values were noticed in the HD subjects. Regression analysis revealed that EMMPRIN and MMP-8 predicted various apoptosis markers, whereas E-cadherin turned out the best predictor of both apoptosis (Hsp27, sFas, sFasL) and matrix turnover (MMP-7, TIMP-1, TIMP-2) indexes in dialyzed patients. Children with CKD are prone to E-cadherin, EMMPRIN and MMP-8 elevation, aggravated by the dialysis commencement and most evident on hemodialysis. Correlations between parameters suggest their role as indexes of apoptosis in children on dialysis. E-cadherin seems the most accurate marker of anoikis in this population.

## Introduction

Enhanced apoptosis is one of the features characteristic for chronic kidney disease (CKD), aggravating with the dialysis commencement and the use of bioincompatible membranes [1, 2]. Anoikis is a specific form of apoptosis, affecting cells that have lost their anchorage in the original milieu [3]. It protects them against migration, reattachment in inappropriate location and metastatic spread. Therefore, anoikis has been studied extensively from the oncologic perspective [3]. Although the essential role in the regulation of cell–cell adhesion has been devoted to integrins, recent investigation has revealed the new players in that puzzle, including cadherins and matrix metalloproteinases [4, 5].

E-cadherin is an adhesion molecule responsible for interactions between neighbouring cells. The loss of its expression is a hallmark of epithelial-mesenchymal transition, changing cell phenotype into the highly invasive one and facilitating metastasis [6]. It has been shown that E-cadherin cleavage may be a caspase-dependent apoptotic process, where the loss of staining on the cell surface is followed by the accumulation of adhesion molecule in the cytosol [7]. E-cadherin can also be a proteolytic target for MMP-7, cleaving an ectodomain with invasive paracrine features [8]. Therefore, like other metalloproteinases, MMP-7 eases cancer invasion by proteolysis of extracellular matrix components and cell detachment.

MMP-7 (matrilysin) is also known for its pro-apoptotic activity, mainly due to the ability of FasL shedding [9, 10]. The similar pro-apoptotic influence has been suggested in the case of MMP-8 (collagenase 2). Indeed, MMP-8 knockout mice suffered from inflammation and concomitant accumulation of neutrophils due to their delayed apoptosis [11].

Another example of an interesting interplay can be observed between MMPs and extracellular matrix metalloproteinase inducer EMMPRIN (CD147/basigin). The latter is able to stimulate MMPs and itself in a paracrine way, aiding tumor angiogenesis. Meanwhile, elevated MMPs can cleave proteolytically a soluble form of EMMPRIN [12].

Despite excessive data concerning the role of afore—mentioned molecules in apoptosis and metastatic spread analyzed separately, little is known about interrelations between them in those processes. None of those elements have been analyzed in CKD or in the view of the dialysis impact on apoptosis either. Having analyzed previously the well-established markers of apoptosis, like Fas/FasL and Hsp27, in combination with gelatinases (MMP-2, MMP-9) in children with CKD [13], we decided to widen the area of interest by examining new markers and testing their potential applicability in the pediatric CKD population.

Therefore, our aim was to assess the serum levels of E-cadherin, EMMPRIN and their potential regulators (MMP-8, MMP-7, TIMP-1, TIMP-2) in children with CKD, on peritoneal dialysis and on hemodialysis, searching for the differences between those modalities and their potential applicability as novel indexes of apoptosis. We also investigated the correlations between those parameters and markers of apoptosis/cell damage (sFas, sFasL, Hsp27) and inflammation (high sensitivity CRP) in the pediatric population with CKD.

## Materials and methods

One hundred and six CKD patients enrolled in the study were divided into four groups. Basic demographic and clinical data are shown in Table 1.

**Table 1:** The basic demographical and clinical data in the groups of CKD, APD and HD children and in controls

Parameter	Median values (interquartile ranges) of analyzed parameters				
	Control gr. (*n* = 30)	CKD I (*n* = 39)	CKD II (*n* = 26)	APD (*n* = 22)	HD (*n* = 19)
Age (years)	10.0 (5.5–15.5)	9.0 (4.5–14.5)	10.5 (2.0–16.5)	10.0 (4.0–15.5)	13.5 (10.5–17.0)
Gender	16 girls; 14 boys	18 girls; 21 boys	10 girls, 16 boys	12 girls; 10 boys	10 girls; 9 boys
Dialysis duration	–	–	–	2.0 years (0.7–2.5)	2.2 years (1.0–2.7)
*Kt*/*V*	–	–	–	1.4 (1.3–1.5)	1.3 (1.2–1.4)
eGFR (ml/min)	101.0 (97.0–110.0)^#^	36.2 (24.3–41.3)^##^	13.0 (10.6-14.8)	–	–
Urea (mg/dl)	35.0 (25.0–42.0)^#^	73.0 (54.0–87.6)^##^	110.0 (98.0–141.0)*	97.4 (81.5–131.4)^•^	135.9 (106.8–161.5)
Albumin (g/dl)	–	4.4 (4.2–4.7)	4.3 (4.1–4.5)	4.3 (4.0–4.5)^•^	3.9 (3.8–4.3)
Hemoglobin (g/dl)	12.0 (11.3–12.9)	11.7 (10.8–13.0)^##^	10.8 (9.9–11.3)*	10.9(9.5–11.9)^•^	9.5 (8.7–10.6)
Parathormone (pg/ml)	–	123.0 (70.0–201.0)^##^	213.5 (98.7–321.5)*	370.5 (50.7–399.8)	290.0 (169.5–904.0)

Mann–Whitney U test: ^#^ *p* < 0.00001 CKD I vs. control group; CKD II vs. control group

^##^ *p* < 0.001 CKD II vs. CKD I

* *p* < 0.00001 CKD II vs. APD, HD; ^**•**^ *p* < 0.00001 APD vs. HD

The first group (CKD I) consisted of 39 patients with CKD stages 3–4 treated conservatively (median GFR calculated according to the Schwartz formula 36 ml/min/1.73 sq m) [14]. The factors causing CKD were: reflux nephropathy (16 cases), chronic glomerulonephritis (12), chronic pyelonephritis (5), polycystic kidney disease (4), haemolytic uremic syndrome (1) and cystinosis (1).

The second group (CKD II) contained 26 patients with CKD stage 5, yet on conservative treatment (median GFR calculated according to the Schwartz formula 13 ml/min/1.73 sq m). Primary diseases causing CKD were: reflux nephropathy (13), chronic glomerulonephritis (9), neurogenic bladder (3), haemolytic uremic syndrome (1). In all patients phosphate binders and vitamin D metabolites were supplemented.

The third group included 22 children on automated peritoneal dialysis (APD—Baxter, Home choice), 5 of them having residual renal function. The patients had 5–8 exchanges of dialysis fluid during the night and, if necessary, one or two during the day. Peritoneal fluids used in our patients had glucose concentrations of 1.36 or 2.27 %. The causative factors in CKD were: chronic pyelonephritis (7), chronic glomerulonephritis (6), polycystic kidney disease (2), neurogenic bladder (3), hemolytic uremic syndrome (2) and unknown (2).

The fourth group consisted of 19 patients hemodialyzed (HD) on polysulfone membranes, only 3 out of them with residual kidney function. HD sessions (3.5–4 h) were performed 3 times a week, using bicarbonate dialysate, the blood flow ranged from 150 to 200 ml/min, dialysate flow did not exceed 500 ml/min. The membrane area was between 1.0 and 1.6 m^2^, the dialyzers were not reused. The water, purified by reosmosis, was regularly checked for contamination. All patients were on stable anticoagulation regimen using non-fractionated or low-molecular-weight heparin. The causative factors in chronic renal failure were: chronic glomerulonephritis (7 cases), chronic pyelonephritis (7), neurogenic bladder (2), polycystic kidney disease (1) and unknown (2).

Thirty children with primary nocturnal enuresis and normal kidney function, served as controls.

None of the patients have shown clinical evidence of infection, had diabetes, malignancies or vasculitides, smoked, took antibiotics, statins, corticosteroids or immunosuppressive therapy. They were also free of such co-morbidities as cardiovascular disease, peripheral vascular disease or obesity. In the CKD group 27 children were normotensive, in 38 patients blood pressure was well controlled with the use of ACE inhibitors (21), calcium channel blockers (10 patients) and β-blockers (3 children), 4 patients needed combined therapy. All APD children had their blood pressure values below the 90th percentile, adjusted for gender, age and height, according to the criteria of the fourth report on high blood pressure in children and adolescents [15] and did not require anti-hypertensives. 7 of our HD patients were normotensive, the rest had their blood pressure well controlled with the use of ACE inhibitors (5), calcium channel blockers (3) or combined therapy with β-blockers (4).

Informed consent was obtained from the subjects and their parents, if necessary. The research project has been approved by the University ethics committee, in accordance with the Helsinki declaration.

Blood samples were drawn after an overnight fast from peripheral veins in CKD and APD patients and in controls, in HD patients from the afferent line of the first-use dialyzer before starting a single session. Samples were clotted for 30 min, centrifuged at room temperature, 1,000 g for 15 min (with the exception of Hsp27, centrifuged at 4 °C), and then serum was stored at −20°C until assayed.

Serum concentrations of E-cadherin, EMMPRIN, MMP-8, Hsp27, sFas, sFasL, MMP-7 (matrilysin), TIMP-1 and TIMP-2 were evaluated by commercially available ELISA kits (Stressgen, R&D Systems, Abingdon, UK). Standards and serum samples were transferred to 96 well microplates pre-coated with recombinant antibodies to human E-cadherin, EMMPRIN, MMP-8, Hsp27, sFas, sFasL, MMP-7, TIMP-1 and TIMP-2. Each sample was tested in duplicate and the arithmetical mean was considered a final result. Measurements were performed according to the manufacturer’s instructions, results were calculated by reference to standard curves.

In all patients high sensitivity CRP (nephelometry by Dade Behring, Marburg, Germany) as a marker of inflammation was also evaluated.

### Statistical analysis

Results are expressed as median values and interquartile ranges. Since the null hypothesis of normality of distribution was rejected by Shapiro–Wilk test, multiple comparisons and comparisons in pairs were evaluated by using nonparametric tests (Kruskall–Wallis, Mann–Whitney U, Wilcoxon). Since the relations between parameters were assessed in the whole group of 65 CKD patients and in the group of all dialyzed patients (APD + HD = 41), in order to get more reliable results, we analyzed the data with the use of Pearson’s correlation coefficient. The statistically significant correlations were then analyzed by linear regression analysis. The linear regression equations were calculated as *y* = β*x* + a (*y*—dependent variable, β—regression coefficient, *x*—independent variable, a–constant term). We presented only those equations where both regression coefficient and constant term were statistically significant. Statistical analysis was performed using the package Statistica ver. 10.0. A *p* value <0.05 was considered significant.

## Results

### E-cadherin, EMMPRIN, MMP-8

**Fig. 1:**
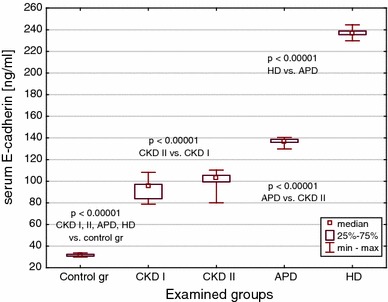
Serum E-cadherin concentrations in the groups of children with CKD, on peritoneal dialysis (APD), on hemodialysis (HD) and in the controls

**Fig. 2:**
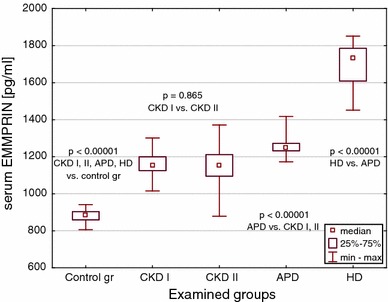
Serum EMMPRIN concentrations in the groups of children with CKD, on peritoneal dialysis (APD), on hemodialysis (HD) and in the controls

**Fig. 3:**
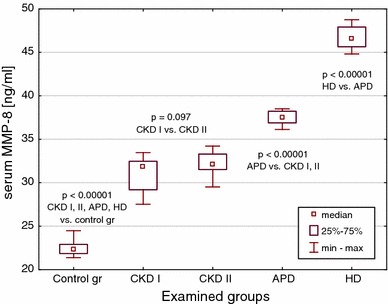
Serum MMP-8 concentrations in the groups of children with CKD, on peritoneal dialysis (APD), on hemodialysis (HD) and in the controls

E-cadherin, EMMPRIN and MMP-8 median values were significantly increased in all CKD children, as well as in all dialyzed patients versus controls (Figs. 1, 2 and 3). The highest concentrations were observed in subjects on hemodialysis. The levels in pre-dialysis children were significantly lower than in those on dialysis, irrespective of the modality. The concentrations of E-cadherin increased proportionately to the renal failure progression (Fig. 1), whereas EMMPRIN and MMP-8 values could not differentiate between CKD stages 3–4 and CKD stage 5 (Figs. 2, 3).

### MMP-7, TIMP-1 and TIMP-2

The median values of MMP-7, TIMP-1 and TIMP-2 were increased in the CKD and dialysis population when compared to controls, and again the highest values were observed in patients on hemodialysis (Table 2). However, there was no significant difference between advanced and end stage renal failure.

**Table 2:** The median values and interquartile ranges of examined parameters in the groups of CKD, APD and HD children and in controls

Parameter	Median values (interquartile ranges) of analyzed parameters				
	Control gr.(*n* = 30)	CKD I (*n* = 39)	CKD II (*n* = 26)	APD (*n* = 22)	HD (*n* = 19)
MMP-7 (ng/ml)	2.2^#^ (2.1–2.3)	2.9 (2.8–3.0)	3.1* (2.4–3.0)	4.16^**•**^ (4.14–4.23)	4.73 (4.59–4.83)
TIMP–1 (ng/ml)	82.3^#^ (80.4–85.1)	130.8 (75.0–135.4)	128.8* (124.3–132.2)	159.2^**•**^ (152.0–163.3)	440.4 (423.6-469.4)
TIMP-2 (ng/ml)	41.9^#^ (39.5–43.5)	130.6 (100.7–144.9)	126.3* (115.3–135.4)	171.95^**•**^ (155.5–176.2)	251.45 (249.3–253.05)
sFas (pg/ml)	3649.15^#^ (3215.81–3721.49)	4943.56^##^ (4623.29–5421.63)	6072.19* (5163.15–6751.0)	9940.39^**•**^ (9812.24–10275.65)	12326.5 (11972.5–12638.4)
sFasL (pg/ml)	43.32^#^ (41.42–44.95)	56.77^##^ (54.75–60.27)	63.71* (60.29–67.46)	88.18^**•**^ (85.65–90.56)	135.06 (133.0–138.28)
Hsp27 (ng/ml)	35.77^#^ (34.16–37.16)	52.4^##^ (49.9–54.58)	56.14* (51.86–58.24)	85.88^**•**^ (84.73–87.43)	115.1 (109.04–120.6)
High sensitivity CRP (mg/l)	0.6 (0.3–1.3)	0.5 (0.2–1.4)	0.4 (0.2–1.5)	0.59 (0.29–1.40)	0.77 (0.2–3.56)

Mann–Whitney U test: ^#^ *p* < 0.00001 CKD I vs. control group; CKD II vs. control group

^##^ *p* < 0.001 CKD II vs. CKD I

*  *p* < 0.00001 CKD II vs. APD, HD

^**•**^ *p* < 0.00001 APD vs. HD

### sFas, sFasL, Hsp27, hsCRP

The median values of sFas, sFasL and Hsp27 were increased in CKD children and in those on dialysis in comparison to the control group, being the highest in patients on hemodialysis (Table 2). hsCRP levels did not differ between examined groups.

### Linear regression analysis

E-cadherin, EMMPRIN and MMP-8 correlated with various metalloproteinases and apoptosis markers in different combinations. Those connections were weaker in the pre-dialysis subjects than in those already on dialysis (Tables 3, 4). In the CKD population none of the above mentioned parameters could show a sufficiently predictive value.

**Table 3:** Correlations between parameters in the group of all CKD children (CKD I + CKD II *n* = 65)

Parameter	MMP-8	EMMPRIN	E-cadherin
MMP-7	*r* = 0.70	*r* = −0.05	*r* = 0.77
	*p* = 0.00001	*p* = 0.68	*p* = 0.00001
MMP-8	–	*r* = 0.30	*r* = 0.83
		*p* = 0.02	*p* = 0.00001
TIMP-1	*r* = 0.77	*r* = 0.41	*r* = 0.83
	*p* = 0.00001	*p* = 0.01	*p* = 0.00001
TIMP-2	*r* = 0.53	*r* = 0.40	*r* = 0.51
	*p* = 0.0001	*p* = 0.01	*p* = 0.0001
E-cadherin	*r* = 0.83	*r* = 0.07	–
	*p* = 0.00001	*p* = 0.58	
Hsp27	*r* = 0.72	*r* = 0.02	*r* = 0.64
	*p* = 0.00001	*p* = 0.85	*p* = 0.0001
sFas	*r* = 0.37	*r* = −0.34	*r* = 0.57
	*p* = 0.01	*p* = 0.01	*p* = 0.0001
sFasL	*r* = 0.51	*r* = 0.15	*r* = 0.70
	*p* = 0.001	*p* = 0.21	*p* = 0.00001
eGFR	*r* = −0.22	*r* = 0.02	*r* = −0.58
	*p* = 0.12	*p* = 0.85	*p* = 0.0001
Urea	*r* = 0.11	*r* = −0.06	*r* = 0.21
	*p* = 0.42	*p* = 0.63	*p* = 0.07
Hemoglobin	*r* = −0.24	*r* = −0.14	*r* = −0.23
	*p* = 0.06	*p* = 0.31	*p* = 0.07
PTH	*r* = 0.0002	*r* = −0.21	*r* = 0.03
	*p* = 0.99	*p* = 0.08	*p* = 0.82

*r* Pearson’s correlation coefficient

**Table 4:** Correlations between parameters in the group of all dialyzed children (APD + HD *n* = 41)

Parameter	MMP-8	EMMPRIN	E-cadherin
MMP-7	*r* = 0.91	*r* = 0.84	*r* = 0.95
	*p* = 0.00001	*p* = 0.00001	*p* = 0.00001
MMP-8	–	*r* = 0.88	*r* = 0.98
		*p* = 0.00001	*p* = 0.00001
TIMP-1	*r* = 0.96	*r* = 0.90	*r* = 0.99
	*p* = 0.00001	*p* = 0.00001	*p* = 0.00001
TIMP-2	*r* = 0.90	*r* = 0.87	*r* = 0.91
	*p* = 0.00001	*p* = 0.00001	*p* = 0.00001
E-cadherin	*r* = 0.98	*r* = 0.98	–
	*p* = 0.00001	*p* = 0.00001	
Hsp27	*r* = 0.95	*r* = 0.88	*r* = 0.96
	*p* = 0.00001	*p* = 0.00001	*p* = 0.00001
sFas	*r* = 0.94	*r* = 0.87	*r* = 0.98
	*p* = 0.00001	*p* = 0.00001	*p* = 0.00001
sFasL	*r* = 0.95	*r* = 0.87	*r* = 0.98
	*p* = 0.00001	*p* = 0.00001	*p* = 0.00001
*Kt*/*V*	*r* = 0.25	*r* = 0.04	*r* = 0.22
	*p* = 0.22	*p* = 0.89	*p* = 0.09
Albumin	*r* = −0.15	*r* = 0.08	*r* = 0.21
	*p* = 0.53	*p* = 0.73	*p* = 0.08
Hemoglobin	*r* = −0.23	*r* = −0.11	*r* = −0.03
	*p* = 0.08	*p* = 0.51	*p* = 0.82
PTH	*r* = 0.05	*r* = 0.19	*r* = −0.12
	*p* = 0.80	*p* = 0.11	*p* = 0.61

*r* Pearson’s correlation coefficient

Contrarily, both EMMPRIN and MMP-8 predicted accurately the values of selected metalloproteinases and apoptosis markers in the group of patients on dialysis (Table 5). However, E-cadherin turned out to be the best predictor of all analyzed parameters.

**Table 5:** The statistically significant correlations between the examined parameters assessed by linear regression analysis in all children on dialysis (APD + HD)

Dependent variable	Independent variable	Regression coefficient β	Constant term	Coefficient of determination *R* ^2^	*p*
Hsp27 (ng/ml)	E-cadherin (ng/ml)	0.30	44.12	0.91	0.00001
sFas (pg/ml)		23.84	6,816.99	0.90	0.00001
sFasL (pg/ml)		0.46	25.65	0.96	0.00001
MMP-7 (ng/ml)		0.01	3.44	0.90	0.00001
TIMP-1 (ng/ml)		2.86	−233.87	0.97	0.00001
TIMP-2 (ng/ml)		0.84	53.23	0.87	0.00001
sFas (pg/ml)	EMMPRIN (pg/ml)	4.52	4,603.81	0.70	0.00001
MMP-7 (ng/ml)		0.01	2.83	0.74	0.00001
TIMP-1 (ng/ml)		0.58	−573.58	0.84	0.00001
TIMP-2 (ng/ml)		0.18	−55.01	0.80	0.00001
MMP-8 (ng/ml)		0.02	14.65	0.81	0.00001
E-cadherin (ng/ml)		0.20	−117.93	0.83	0.00001
Hsp27 (ng/ml)	MMP-8 **(**ng/ml)	3.17	−32.82	0.90	0.00001
sFasL (pg/ml)		4.73	−87.08	0.90	0.00001
MMP-7 (ng/ml)		0.06	2.08	0.84	0.00001
TIMP-1 (ng/ml)		30.23	−973.94	0.94	0.00001
TIMP-2 (ng/ml)		8.88	−164.31	0.84	0.00001
E-cadherin (ng/ml)		10.55	−257.33	0.96	0.00001

No significant associations between examined parameters and hsCRP were observed. Only E-cadherin correlated inversely with eGFR. No correlations with selected parameters of dialysis adequacy, such as *Kt*/*V*, hemoglobin, albumin, urea, or calcium-phosphate metabolism (parathormone) were noticed either (Tables 3,  4).

## Discussion

Our study has shown for the first time the elevation of E-cadherin, EMMPRIN and MMP-8 concentrations in children with CKD, pointing at their potential role as markers of apoptosis in the patients with different stages of CKD.

E-cadherin serum levels were higher in pre-dialysis patients than in controls and increased progressively with renal failure aggravation. Since there are no other observations of sE-cadherin in CKD patients, irrespective of their age, we could only hypothesize on the cause of such elevation. The first explanation, strengthened by the inverse correlation between sE-cadherin and eGFR, is the molecule accumulation due to decreasing glomerular filtration. This connection would also explain the statistically significant difference between values in CKD stages 3–4 and stage 5. Another reason for sE-cadherin elevation could be a direct consequence of the way in which that adhesion molecule is released. MMPs, with MMP-7 above all, are known to transform the membrane-anchored E-cadherin into its circulating form [8]. The correlations we noticed between E-cadherin and all analyzed MMPs could be an indirect proof for such dependence in CKD children. MMP overactivity in CKD patients has already been studied, thus explaining its potential impact on E-cadherin release [16].

The E-cadherin proteolysis results in disintegration of cell–cell junctions, giving way to cell detachment. The latter triggers anoikis, preventing those cells from reattachment and dysplasia. Within normal conditions, this mechanism guarantees the balance between survival and death. However, when MMP concentrations are increased and their proteolytic overactivity results in massive E-cadherin loss, the resistance to anoikis and increased angiogenesis are granted [6]. Thus, soluble E-cadherin could serve as a direct marker of anoikis intensity in this population of patients.

The situation is even more evident in the subjects on dialysis—sE-cadherin concentrations were higher in that group than in the pre-dialysis children. Similarly to the case of MMPs, that increase was more obvious in children on hemodialysis, confirming the larger activity of apoptotic processes in the group of patients undergoing this type of renal replacement therapy. Likewise, correlations of sE-cadherin with MMPs and markers of apoptosis were more significant in patients on dialysis than in pre-dialysis children and were additionally strengthened in the dialyzed patients by their significantly predictive value. The main underlying cause of such differences might be the dialysis commencement, whereas hemodialysis–peritoneal dialysis discrepancy may result from greater bioincompatibility of materials used in hemodialysis and a direct cell detachment provoked by blood-membrane contact. The toxicity of uremic serum itself, together with sinusoidal changes in toxin concentrations between hemodialysis sessions, create unfavorable conditions, under which cells are more prone to apoptosis. Peritoneal dialysis, a method of choice in smaller children, seems less vulnerable, mainly due to its continuity and better metabolic control.

The discrepancy in sE-cadherin concentrations and the strength of correlations with MMPs and apoptosis markers, observed between pre-dialysis and dialyzed subjects, may also have an anxious context. The cut-off point for E-cadherin loss intensity, distinguishing between anoikis and anoikis resistance, is not known. From that point of view, every factor intensifying this process, like MMP overactivity, may cause a pro-metastatic breakthrough. Patients on hemodialysis are at greater risk of tumorigenesis than the healthy population. Therefore, sE-cadherin could serve as an emerging danger signal in screening those patients. However, such hypotheses require verification in population-based studies.

EMMPRIN serum concentrations were significantly increased in CKD pre-dialysis children when compared to controls, although they failed to differentiate between CKD stages 3–4 and stage 5. The significant increase was noticed when the children on dialysis were analyzed, with the highest values in HD patients. No data on EMMPRIN in CKD patients are available, so we carefully hypothesized that molecule accumulation could be of importance in this process. However, since no correlation with eGFR was noticed, other factors, like dialysis procedure, might play more important role in this increase than the uremia itself.

Indeed, we have found few borderline correlations of EMMPRIN with TIMP-1 and sFas in CKD children, whereas in the dialysis group EMMPRIN became a good predictor of all MMPs, TIMPs and apoptosis markers. Therefore, renal replacement therapy itself must be treated as a factor increasing the concentrations of analyzed parameters, thus confirming the progressing vulnerability towards apoptosis. The interesting aspect is the already mentioned impact that EMMPRIN exerts on MMPs and TIMPs [12]. It is probable that the concentrations of analyzed proteolytic enzymes remained fairly stable during late stages of CKD due to the fact that EMMPRIN concentrations behaved in a similar way.

There is also, like in the case of E-cadherin, a dangerous aspect of tumorigenesis that EMMPRIN is known to stimulate [17]. The only available data on increased soluble EMMPRIN concentrations in nephrology concern patients with urothelial carcinoma of the bladder [18]. When the fact that significant correlations between EMMPRIN and apoptosis markers are only seen in the population on dialysis, is taken into account, it seems probable that the therapy itself adds to the uremic milieu, creating conditions where the MMP inducer becomes a valuable marker of apoptotic processes.

Serum MMP-8 followed the same concentration pattern as EMMPRIN, once again showing significant differences in CKD patients versus controls, pre-dialysis versus dialysis subjects and hemodialysis versus peritoneal dialysis. Apart from the previously discussed possible explanations, such as molecule accumulation and further negative dialysis impact, there is an aspect typical for MMP-8. This enzyme is released from the granules of polymorphonuclear leukocytes. The triggering factors might be proinflammatory cytokines, such as IL-1 or TNF-α, that are upregulated in the course of CKD. The additional impulse for MMP-8 release may be the direct blood-membrane contact with subsequent transient leukocytosis during hemodialysis sessions, explaining the difference between HD and APD patients. It is known from the in vitro studies that MMP-8 release is a direct inducer of endothelial cell apoptosis [19]. The value of this metalloproteinase as a potential marker of apoptosis has been confirmed by our results. We found significant correlations between MMP-8, sFas/sFasL and Hsp27, more evident in the dialyzed patients. Moreover, MMP-8 turned out a valuable predictor of those parameters in children on dialysis. Significant correlations with serum E-cadherin and EMMPRIN suggest that this collagenase may also regulate in turn, by proteolysis, the activity of both adhesion molecule and MMP inducer, thus controlling in an indirect way the intensity of apoptosis.

Finally, we are aware of the limitations of our study that have to be acknowledged. Due to the lack of comparative data and reference values, both in adult and pediatric patients, we have to be cautious in drawing conclusions. The selected parameters we assessed do not picture the whole complexity of interactions between sE-cadherin, EMMPRIN, MMP-8, Fas/FasL and Hsp27, either explain fully their impact on apoptosis intensity. This fascinating subject we have just started exploring definitely needs further investigation performed on a larger group of patients.

## Conclusions

EMMPRIN, E-cadherin and MMP-8 elevation, observed in children with CKD, is aggravated by the dialysis commencement and more pronounced in patients on hemodialysis. Correlations between examined parameters suggest their potential role as indexes of apoptosis in children on chronic dialysis, pointing specifically at E-cadherin as the most reliable marker of anoikis in this population.
